# Does Zika Virus Really Causes Microcephaly in Children Whose Mothers Became Infected with the Virus during Their Pregnancy?

**Published:** 2018-04

**Authors:** Pendru RAGHUNATH

**Affiliations:** Dept. of Microbiology, School of Medicine, Texila American University, Georgetown, Guyana

## Dear Editor-in-Chief

Zika virus is an enveloped, positive-strand RNA flavivirus. It was first isolated in 1947 from the Zika forest of Uganda from a Rhesus macaque. After that for many years, it was not known whether the virus can cause human disease. The epidemiology of Zika virus changed since 2007 when an outbreak occurred on Yap Island of the Federated States of Micronesia. Zika virus infection generally leads to self-limiting mild, febrile illness. However, many of the recent outbreaks were linked to upsurge in cases of GuillanBarré syndrome (GBS) and a rise in infants born with microcephaly. Placenta and amniotic fluid of women with microcephalic fetuses and blood of neonates with microcephaly were found positive for Zika virus RNA suggesting the transplacental transmission of Zika virus. The virus has also been identified in the brains and retinas of microcephalic fetuses (1).

Despite accumulating clinical evidence, direct experimental evidence showing that the Zika virus causes birth defects remains absent. Zika virus infects fetuses, is causing intrauterine growth restriction, including signs of microcephaly, in mice (1). Zika virus infects human cortical progenitor cells in vitro, leading to an increase in cell death (1). Zika virus efficiently infects organoids and causes a decrease in overall organoid size through activation of the innate immune receptor Toll-like-receptor 3 (TLR3) (2). Nowakowski and colleagues found that AXL is highly expressed by human radial glial cells, astrocytes and microglia in developing human cortex (3). They also proposed AXL as a candidate viral entry receptor and Zika virus reaches the developing brain and invades radial glial cells with highest AXL expression (3). By preferentially destroying radial glial cells, Zika virus can produce severe microcephaly. Evolutionary changes such as mutations or recombination events might be responsible for the increased virulence and a new spectrum of Zika disease. Recombination events were reported to occur in different Zika viral strains. The distinct amino acid variations were reported in the structural and nonstructural proteins of all Zika virus strains responsible for 2015–2016 outbreaks.The role of those variations in the virulence of Zika virus or host-virus interaction needs to be studied. In the past two decades, dengue virus has spread through areas of South America, and the seroprevalence of dengue in some Zika virus affected areas exceeds 90%. Many reports demonstrated a degree of antigenic similarity between the dengue and Zikaviruses. Antibody-dependent enhancement (ADE) of infectionis common among Dengue virus serotypes. Dengue antibodies were able to bind Zika virus but were unable to neutralize the virus and instead promoted ADE (4). Hence, immunity to dengue virus might enhance replication of Zika virus and play an important role in pathogenesis and disease outcome in Zika infection.

Zikavirus and man share a peptide in common, hence, immune response to Zika virus might cause damage to host tissue and responsible for all the neurological complications (5). Along with that, there could be other associated risk factors/agents responsible for microcephaly. Protein extracts of three Zika positive brains were analyzed by shotgun mass spectrometry and the presence of peptide(s) from the polyprotein of a Bovine-like viral diarrhea virus (BVDV) was reported (6). However, BVDV is not known to cause disease or even infection in humans. It is also a known contaminant in many cell culture reagents. Of the 25 identifications of BVDV derived peptides, 24 turned out to be identical to human proteins, many from the ubiquitin-family. Hence, their findings are of doubtful significance and need experimental validation.

## References

[1] Cugola FR Fernandes IR Russo FB (2016). The Brazilian Zika virus strain causes birth defects in experimental models. Nature, 534:267–271. 27279226 10.1038/nature18296 PMC4902174

[2] Dang J Tiwari SK Lichinchi G (2016). Zika Virus Depletes Neural Progenitors in Human Cerebral Organoids through Activation of the Innate Immune Receptor TLR3. Cell Stem Cell, 19(2): 258–265. 27162029 10.1016/j.stem.2016.04.014 PMC5116380

[3] Nowakowski TJ Pollen AA Di Lullo E (2016). Expression Analysis Highlights AXL as a Candidate Zika Virus Entry Receptor in Neural Stem Cells. Cell Stem Cell, 18(5): 591–596. 27038591 10.1016/j.stem.2016.03.012 PMC4860115

[4] Dejnirattisai W Supasa P Wongwiwat W (2016). Dengue virus sero-cross-reactivity drives antibody-dependent enhancement of infection with zika virus. Nat Immunol, 17(9): 1102–1108. 27339099 10.1038/ni.3515 PMC4994874

[5] Lucchese G Kanduc D (2016). Zika virus and autoimmunity: from mycrocephaly to Guillain-Barre syndrome, and beyond. Autoimmun Rev, 15(8):801–808. 27019049 10.1016/j.autrev.2016.03.020

[6] Nogueira FCS Velasquez E Melo ASO Domont GB (2016). Zika virus may not be alone: proteomics associates a bovine-like viral diarrhea virus to microcephaly. Biorxiv preprint first posted online Jul. 15, 2016.

